# Towards conformational fidelity of a quaternary HIV-1 epitope: computational design and directed evolution of a minimal V1V2 antigen

**DOI:** 10.1093/protein/gzy010

**Published:** 2018-06-12

**Authors:** Jennifer I Lai, Deeptak Verma, Chris Bailey-Kellogg, Margaret E Ackerman

**Affiliations:** 1 Thayer School of Engineering, Dartmouth College, 14 Engineering Dr, Hanover NH, USA; 2 Department of Computer Science, Dartmouth College, 9 Maynard St, Hanover NH, USA; 3 Department of Microbiology and Immunology, Geisel School of Medicine, Dartmouth College, 1 Medical Center Dr, Lebanon NH, USA

**Keywords:** computational protein design, directed evolution, epitope-focusing, HIV-1, structure-based vaccine design

## Abstract

Structure-based approaches to antigen design utilize insights from antibody (Ab):antigen interactions and a refined understanding of protective Ab responses to engineer novel antigens presenting epitopes with conformations relevant to eliciting or discovering protective humoral responses. For human immunodeficiency virus-1 (HIV-1), one model of protection is provided by broadly neutralizing Abs (bnAbs) against epitopes present in the closed prefusion trimeric conformation of HIV-1 envelope glycoprotein, such as the variable loops 1–2 (V1V2) apex. Here, computational design and directed evolution yielded a novel V1V2 sequence variant with potential utility for inclusion in an immunogen for eliciting bnAbs, or as an epitope probe for their detection. The computational design goal was to engineer a minimal single-chain antigen with three copies of the V1V2 loops to support maintenance of closed prefusion V1V2 trimeric conformation and presentation of bnAb epitopes. Via directed evolution of this computationally designed single-chain antigen, we isolated a V1V2 sequence variant that in monomeric form exhibited preferential recognition by quaternary-preferring and conformation-dependent mAbs. Structural context and transferability of this phenotype to V1V2 sequences from all strains of HIV-1 tested suggest a conformation-stabilizing effect. This example demonstrates the potential utility of computational design and directed evolution-based protein engineering strategies to develop minimal, conformation-stabilized epitope-specific antigens.

## Introduction

Structure-based design approaches for next-generation immunogens and probes utilize structural information of antibody (Ab):antigen recognition to present epitopes against which particularly potent or broadly neutralizing Ab (bnAb) responses develop (Kulp and Schief, 2013; McLellan *et al.*, 2013; Correia *et al.*, 2014; Impagliazzo *et al.*, 2015; Rappuoli *et al.*, 2016). As the conformational state of an epitope may relate to its cognate Ab’s neutralization potency (Sanders *et al.*, 2013), novel immunogens and probes require (i) understanding the structural determinants of epitopes recognized by protective Abs, and (ii) selective presentation of the epitope(s) of interest in conformations relevant to recognition by bnAbs. Successful design solutions to this protein engineering problem will result in novel antigens that may serve as promising vaccine immunogens to elicit desired immune responses and/or as epitope probes to characterize humoral responses or isolate novel Abs against epitopes of interest. Furthermore, a focus on minimal antigens—constructs encompassing residues that comprise an epitope and are necessary for maintaining its proper conformation—holds promise to selectively elicit potent humoral responses, which may be crucial in cases where irrelevant immunodominant epitopes distract from vulnerable epitopes. Minimal antigens may also afford epitope-specific humoral profiling or the isolation of novel Abs with unprecedented epitope and conformational specificity.

The human immunodeficiency virus-1 (HIV-1) variable loops 1–2 (V1V2 loops) on the envelope glycoprotein (gp120) comprise a promising antigenic site for vaccine targeting and humoral profiling; the V1V2 loops, despite high sequence variability, are structurally and functionally conserved (Zolla-Pazner and Cardozo, 2010), and are targeted by an extended class of highly potent V1V2-targeting bnAbs, of which PG9 and PG16 are prototypical examples (Walker *et al.*, 2009; McLellan *et al.*, 2011). Furthermore, anti-V1V2 loop Abs directed against the V1V2 loops were associated with vaccine-mediated protection in the RV144 trial (Haynes *et al.*, 2012; Gottardo *et al.*, 2013; Zolla-Pazner *et al.*, 2014). The PG9 class of bnAbs preferentially recognizes the V1V2 loops as presented on the HIV-1 envelope trimer and demonstrates limited reactivity to monomeric gp120 from some HIV-1 strains (Walker *et al.*, 2009; McLellan *et al.*, 2011; Julien *et al.*, 2013b), while other V1V2-targeting monoclonal Abs (mAbs) such as 697-30D and CH58/CH59 recognize distinct conformational and linear V2 epitopes, respectively (Gorny *et al.*, 1994, 2012; Liao *et al.*, 2013). That there exist distinct classes of anti-V1V2 loop mAbs recognizing quaternary, conformational or linear epitopes overlapping in sequence suggests that the V1V2 loops may exist dynamically depending on their molecular context within a monomer or trimer (Alter and Ackerman, 2013).

Given this metastability of V1V2 structure, a more nuanced understanding of the conformational reactivity in anti-V1V2 humoral responses when characterizing or isolating mAbs from HIV-infected subjects or vaccine recipients may provide further insights into structural determinants of protective versus non-protective responses. For instance, characterization of Ab responses in RV144 vaccinees showed differential sera reactivity to vaccine prime and boost components [canary-pox-vectored ALVAC‐HIV (vCP1521) incorporating the envelope glycoprotein gene and recombinant AIDSVAX gp120 B/E protein, respectively], which was speculated to result from differential trimeric versus monomeric gp120 conformations (Alter and Ackerman, 2013; Liao *et al.*, 2013). While protection in the RV144 trial was correlated with Ab responses to the V1V2 loops as measured by titers against a gp70-scaffolded V1V2, mAbs like CH58 and CH59 isolated from RV144 vaccinees were shown to recognize linear epitopes and possessed narrow neutralization activity (Liao *et al.*, 2013). Thus, while the use of currently available epitope-specific probes has enabled epitope-specific dissection of humoral responses to HIV-1 and the isolation of mAbs from subject samples (Azoitei *et al.*, 2011; Wu *et al.*, 2010), a probe enabling more fine-grained characterization with respect to epitope conformation has the potential to deepen understanding of humoral responses desired from vaccination.

The goal for this study was to engineer a minimal HIV-1 antigen for use as a V1V2 loop immunogen or probe. Given the importance of trimeric envelope conformation, we aimed to display the V1V2 loops with conformational fidelity to their trimeric presentation in the full-length prefusion envelope trimer, yet expressed as a single protein chain: a single-chain trimeric V1V2 [sc-(V1V2)^3^]. Our approach combined computational protein design utilizing HIV-1 prefusion envelope structural information (Julien *et al.*, 2013a) as a starting blueprint, and directed evolution of a yeast surface-displayed sc-(V1V2)^3^ library with known V1V2-targeting mAbs (Table I) as selection reagents for desired conformational properties, to engineer a minimal antigen with conformation-restricted display of the V1V2 loops. An initial computational sc-(V1V2)^3^ design was experimentally determined to show modest reactivity to quaternary-preferring and conformational mAbs, suggesting promising epitope conformation. Further optimization via directed evolution resulted in a truncated design, thus falling short of the initial design goal, yet nonetheless yielded a V1V2 sequence variant with enhanced recognition by trimer-preferring or conformational mAbs over those recognizing linear epitopes in the context of multiple V1V2 antigens and HIV-1 strains. As a single point mutation, this sequence variant thus moves towards presentation of the V1V2 loops with conformational relevance to bnAb recognition in an epitope-specific probe. Use of this variant as a probe may afford profiling V1V2-specific responses or isolating novel mAbs with finer resolution towards conformational specificity, and as an immunogen may have greater potential to elicit conformation-restricted Ab responses. Furthermore, this result demonstrates that even in the context of sequence-hypervariable viral proteins, protein engineering techniques such as directed evolution can be successfully applied to epitope-focused immunogen design in cases where conformational epitope presentation is key to eliciting protective humoral responses.

**Table I.** Human V1V2 mAbs used in this study

Recognition mode	mAbs	Neutralization potency	Epitope and structure specificity
Quaternary-preferring^a^	PG9, PG16	+++	Backbone contacts, glycan-dependent
Conformational^b^	697-30D	~Tier 1 viruses	Conformational discontinuous epitope
Peptide^c^	CH58, CH59	Lab-adapted viruses	Linear epitope, helix/coil secondary structure

^a^Walker *et al.*, 2009; McLellan *et al.*, 2011.

^b^Gorny *et al.*, 1994; Zolla-Pazner *et al.* 1995; Israel *et al.*, 1997.

^c^Bonsignori *et al.*, 2012; Liao *et al.*, 2013.

## Materials and Methods

### General

Molecular biology reagents: DNA for sc-(V1V2)^3^ and gp70 V1V2 constructs were purchased as gBlock gene fragments from Integrated DNA Technologies (IDT), or synthesized by Genewiz, Inc. All oligonucleotides were purchased from IDT. Phusion DNA Polymerase, Taq polymerase, DNA restriction and ligation enzymes were purchased from New England Biolabs (NEB), and DNA purification and gel-extraction were performed using Geneaid kits. Site-directed mutagenesis was performed with Turbo pfu polymerase (Agilent). DNA sequences were verified by Genewiz, Inc. *Escherichia coli* strains DH5α, 10-β electrocompetent (NEB) were used as hosts for molecular cloning. DNA plasmid purification from bacteria was performed using QIAPrep Spin Miniprep (Qiagen), Zyppy Plasmid Midiprep (Zymo Research) or E.Z.N.A. Plasmid Mega kits (Omega Bio-tek). DNA purification from yeast was performed using Zymoprep Yeast Plasmid Miniprep II kit (Zymo Research).

Antibodies: The following reagents were obtained through the NIH AIDS Reagent Program, Division of AIDS, NIAID, NIH: CH58 and CH59 from Drs Barton F. Haynes and Hua-Xin Liao (Bonsignori *et al.*, 2012; Liao *et al.*, 2013); anti-HIV-1 gp120 Monoclonal (697-30D) from Dr. Susan Zolla-Pazner (Gorny *et al.*, 1994; Zolla-Pazner *et al.*, 1995; Israel *et al.*, 1997); anti-HIV-1 gp120 Monoclonal (PGT145) (Walker *et al.*, 2011); catalog #3957, HIV-IG from NABI and NHLBI. Antigens: Commercially available gp70 V1V2 AE.A244 (IT-001-212p) and gp120 CM235 (IT-001-150p) were acquired from Immune Technology Corp.

Heavy and light variable region DNA for PG9 and PG16 were purchased as gBlocks (IDT) and cloned into CMVR vectors. Anti-DENV EDE B7 in the pMAZ heavy and light chain vectors was generously provided by Jonathan Lai (Einstein College of Medicine).

Cell lines: Yeast strain *Saccharomyces cerevisiae* EBY100 was used for yeast surface display (Boder and Wittrup, 1997), and HEK293F suspension cells were used for both mammalian soluble and surface expression.

### Computational protein design

**Fig. 1:**
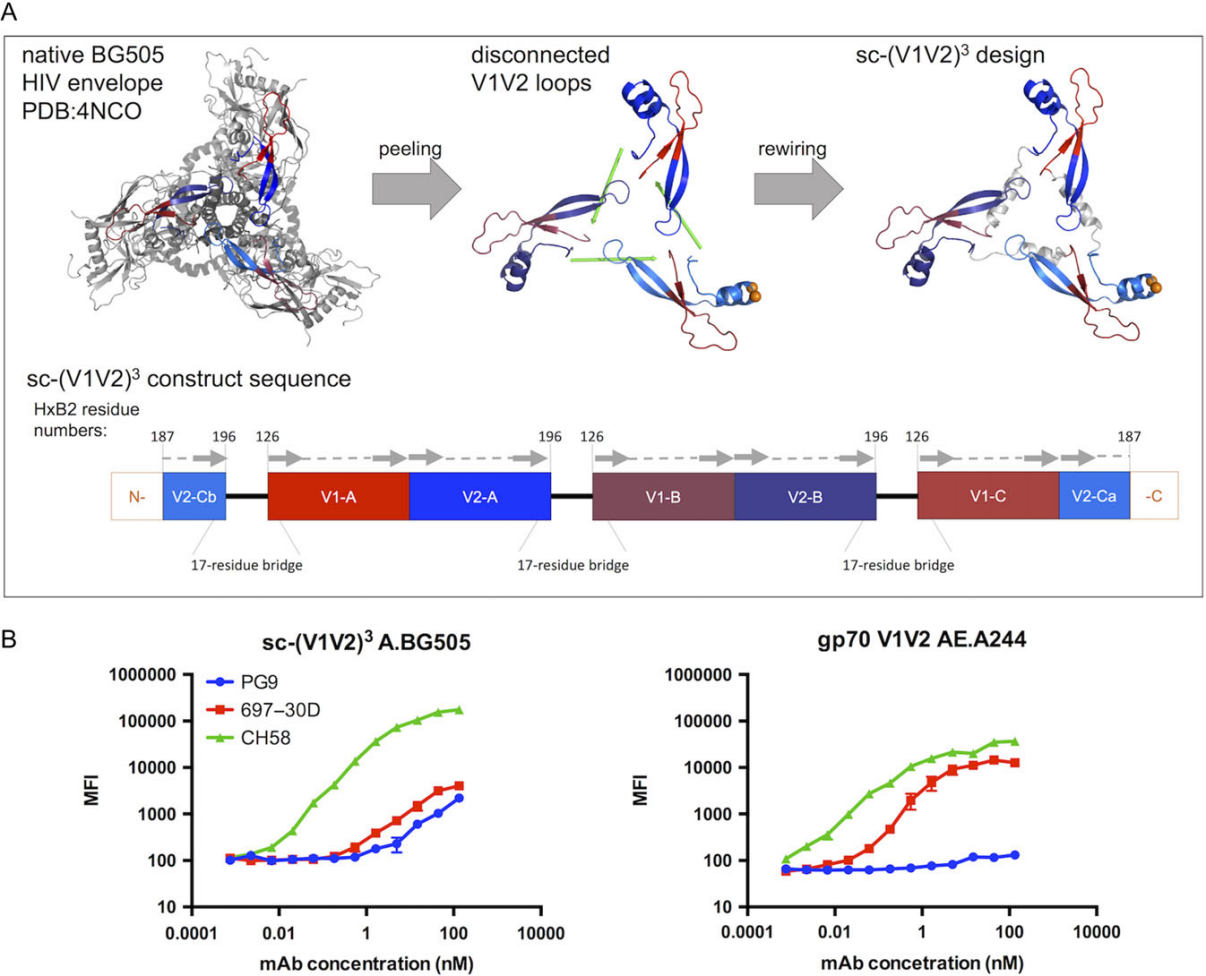
Computational design and characterization of sc-(V1V2)^3^. (**A**) Top view of the native BG505 HIV envelope trimer used as a starting point for the computational sc-(V1V2)^3^ design (Left). A protein peeling algorithm identified V1V2 loop boundaries (Center), and computational rewiring (designation of new N- and C- termini and design of helical bridges to connect V1V2 loop segments) resulted in the sc-(V1V2)^3^ design (Right). Expression order is depicted schematically to show distinct V1 and V2 loops (A–C), the corresponding residues (HxB2 numbering), the length of the introduced residue bridges, as well as the V1V2 breakpoint introduced by the novel termini (V2-Ca/V2-Cb). V1V2 loop secondary structure elements (McLellan *et al.*, 2011) are schematized by arrows (β-strands) or dashed lines (loops). (**B**) HEK293-secreted sc-(V1V2)^3^ was assessed for binding to CH58, 697-30D and PG9, and the relative binding profile was compared to that observed for gp70 V1V2 AE.A244. Error bars represent standard deviation of triplicate measurements.

Computational design of sc-(V1V2)^3^ was performed via the following steps (Fig. 1): (i) extracting the trimeric V1V2 epitope region, (ii) constructing a single protein chain connecting discontiguous fragments and (iii) stabilizing this antigen.

- The three V1V2 loops were extracted from the BG505 prefusion envelope trimer crystal structure ((Julien *et al.*, 2013a), PDB ID 4NCO) using a protein peeling algorithm (Gelly *et al.*, 2006; Gelly and de Brevern, 2011) with default parameters. Briefly, the algorithm hierarchically divides a structure into well-organized, compact units by maximizing intra-unit and minimizing inter-unit Cα-Cα contact distances, yielding sub-domain protein units (here, three separate V1V2 loops).
- The three extracted V1V2 loops in their trimeric conformation were re-ordered and re-wired into a single chain. New termini were identified by scanning a single V1V2 loop to identify a position predicted to permit introduction of new termini. In particular, contact residues within 8 Å for each residue were averaged. Residue 188 in the BG505 V1V2 loop located in the hypervariable region of V2 was identified as having minimal averaged contacts, and thus, residues 189 G and 188Q (BG505 numbering; between 187 and 188 in HxB2 numbering) were selected as new N- and C-termini, respectively. The MASTER algorithm (Zhou and Grigoryan, 2015) was employed to identify appropriate bridging fragments to connect the V1V2 loops, searching a library derived from existing PDB structures while ensuring proper distance and backbone fit to reduce the likelihood of clashes within the resulting protein.
- Modeling of missing regions of V2 from the BG505 SOSIP structure, and bridge redesign by mutation were performed with RosettaRemodel (Huang *et al.*, 2011). From many possible designs, an sc-(V1V2)^3^ with best Rosetta energy was selected. A full water molecular dynamics simulation was performed with Gromacs (Berendsen *et al.*, 1995) to check for abnormal dynamic behavior, such as high fluctuations of the V1V2 loops or unfolding of the inserted bridges. The full sc-(V1V2)^3^ sequence is available in Supplemental Fig. S2.

### Preparation of DNA constructs

The sc-(V1V2)^3^ protein sequence with C-terminal avi and 6xHis tags was mammalian codon optimized, synthesized and cloned into a CMVR vector. For yeast surface display, sc-(V1V2)^3^ was inserted between restriction sites NheI and BamHI in the yeast pCT vector. For sc-(V1V2)^3^ clone 1.3.9 revertants, pCT plasmid containing clone 1.3.9 was first isolated via Zymoprep, amplified in bacterial culture (NEB 10-β electrocompetent cells) and purified via miniprep. Site-directed mutagenesis was performed following the QuikChange site-directed mutagenesis protocol (Agilent). Expression in HEK membrane display was achieved by cloning sc-(V1V2)^3^ into the ppPI4 vector, resulting in a C-terminal gpi anchor motif. Mutant sc-(V1V2)^3^ was generated via site-directed mutagenesis as described above.

The gp70 V1V2 constructs for strain BG505 were mammalian codon optimized and cloned into a CMVR vector. Constructs for gp70 V1V2 from strains CaseA2, 92Th023 and CM244 were synthesized by Genewiz, Inc., in the CMVR vector backbone, and with C-terminal Avi and 6xHis tags. Constructs were cloned into the ppPI4 surface display vector, and K-M or R-M mutants were constructed via site-directed mutagenesis as described above.

### Mammalian protein production and purification

HEK293F cells were transiently transfected with secreted protein construct expression vectors [sc-(V1V2)^3^ and gp70 V1V2/gp70 scaffold only in CMVR plasmids] with polyetheleneimine (PEI) (Polysciences 23966), and cultured for 5–7 days. Prior to purification, cultures were centrifuged at 9000 *g* for 15 min and filtered using 0.22 μm Steritop filter units (Millipore). Proteins were purified using ion metal affinity chromatography (IMAC) using a nickel-charged IMAC Sepharose 6 Fast Flow column followed by size exclusion chromatography using a HiLoad 16/600 Superdex 75 pg column on an AktaPure system (GE Healthcare Life Sciences).

### Customized microsphere immunoassays

Antibody binding to sc-(V1V2)^3^ and gp70 V1V2 BG505 WT and K-M mutants was assessed via a customized immunoassay as previously described (Brown *et al.*, 2012). Briefly, proteins were conjugated to magnetic carboxylated fluorescent beads (Luminex Corp) using a two-step carbodiimide reaction. Proteins conjugated to beads were also deglycosylated with PNGaseF treatment as previously described (Brown *et al.*, 2017): conjugated beads were buffer exchanged into 20 mM Tris pH 8.2 to a final concentration of 100 beads per type per μL. PNGaseF enzyme (NEB P0704S) was added at a concentration of 10 000 U PNGaseF/1000 000 beads, and incubated overnight at 37°C with rotation. PNGaseF treated beads were buffer exchanged into Assay Buffer (PBS + 0.1% BSA + 0.05% Tween-20) prior to use. As the Luminex platform allows for multiplexing, 500 beads/well of each type of antigen-coated bead was incubated with 50 μL primary antibody or biotinylated Galanthus Nivalis Lectin (Vector Labs B-1245) at the desired final concentration in 384-well polystyrene microplates (Greiner Bio-One) for 1 h at room temperature in Assay Buffer. Plates were then washed five times in Assay Buffer using a BioTek plate washer. Beads were then incubated in secondary antibody—anti-human Fc-PE, anti-human lambda-PE, anti-human kappa-PE, or streptavidin-conjugated PE (Southern Biotech 2048-09, 9180-09, 2060-09, 7100-09 M)—at a concentration of 0.65 μg/mL in Assay Buffer for 1 h. All incubations were performed on a Microtiter Shaker (VWR). A final wash was conducted with xMAP Sheath Fluid (Luminex), and beads were resuspended in 40 μL Sheath Fluid prior to running assay plates on a Bio-Plex array reader (FlexMAP 3D, Luminex). Median fluorescence intensities were reported for each bead type, and secondary only controls were used to assess background fluorescence levels.

### Yeast surface display and flow cytometric analysis

EBY100 were transformed with the pCT sc-(V1V2)^3^ yeast display construct using the Frozen-EZ yeast Transformation II Kit (Zymo Research) according to the manufacturer’s instructions. Clones were grown on SDCAA plates at 30°C, and colonies were then used to inoculate SDCAA medium overnight. For induction of protein expression, a pre-culture was grown overnight at 30°C, and a fresh culture was started at OD600 of 0.2. At OD600 of 1, the culture was transferred to SGCAA media, and grown overnight at 30°C.

Induced cells were analyzed by flow cytometry on a MACSQuant Analyzer (Miltenyi Biotec). Briefly, 1.5–2.5e5 cells were washed in PBSF (PBS + 0.1% BSA) and incubated in 50 μL of primary antibody at desired concentration and a 1:200 dilution of chicken anti-c-myc (Gallus Immunotech) in PBSF for 1 h at room temperature in 96-well conical bottom plates (USA Scientific). Cells were centrifuged and washed three times in PBSF before incubation in secondary antibodies, goat anti-chicken IgY (H + L) Alexa Fluor 488 and goat anti-human (H + L) Alexa Fluor 647 (A-11039, A-21445 ThermoFisher), at a 1:1000 dilution in PBSF for 20 min at room temperature. All incubations were performed on a Microtiter Shaker (VWR). Prior to analyzing samples, cells were washed two times in PBSF and resuspended in 200 μL PBSF.

For analysis, cells were gated first using forward and side scatter to exclude debris. Samples were then gated for expression based on comparison to a secondary-only control—high anti-c-myc 488 signal—and the median fluorescence intensity of the anti-human 647 signal for this ‘expressing’ population was used as a measure of antibody binding.

### Yeast library construction

The sc-(V1V2)^3^ library was prepared essentially as described previously (Chao *et al.*, 2006; Grimm *et al.*, 2015). Inserts for an sc-(V1V2)^3^ yeast display library were constructed by error-prone PCR (epPCR) as previously described (Fromant *et al.*, 1995), aiming for four amino acid mutations per gene. Products from this epPCR reaction were purified (Geneaid) and subjected to a second round of standard amplification using Phusion polymerase. Vector (250 μg of pCT) was prepared by restricting with BamHI-HF and NheI-HF (NEB). Vector was gel extracted using a DNA Extraction Maxi Kit (Geneaid), and both insert and vector were ethanol-precipitated with Pellet Paint (Millipore), and resuspended in water. A total of 600 μL of electrocompetent *S. cerevisiae* EBY100 were prepared as described previously (Chao *et al.*, 2006), mixed with all insert and vector, and split into four 2 mm cuvettes. Cells were transformed at 1.2 kV and 25 μF using a Gene Pulser Xcell (Bio-Rad), recovered in a 1:1 mixture of 1 M sorbitol:YPD at 30°C for 1 h. Cells were transferred to 1 L SDCAA media, and serial dilutions were plated on SDCAA plates for 2 days at 30°C to estimate library diversity.

### Directed evolution magnetic and fluorescence activated selection

For each round of library selection and assessment, the library was induced by starting a subculture at 0.2 OD600 at a volume such that library diversity was oversampled by at least 10-fold. Yeast were induced by switching into SGCAA media and incubating at 30°C overnight.

The first two selections (resulting in generations 1.1. and 1.2) were performed using magnetic activated cell sorting (MACS) (Ackerman *et al.*, 2009). Briefly, magnetic Dynabeads Protein A (ThermoFisher) were coated with a saturating concentration of selection Ab (determined by titration) on a rotator for at least 1 h at 4°C. Prior to adding beads to the yeast library, beads were washed on a magnet a minimum of five times with 1 mL PBSF to remove excess antibody. For each selection step, the library was incubated with beads in 1 mL PBSF and incubated with rotation for 1.5 h at 4°C. Following selection, beads were washed and incubated in 1 mL PBSF with rotation at 4°C for 15–30 min. Beads were resuspended in PBSF and saved as the ‘bead fraction’, and wash supernatants were saved as the ‘wash fraction’. Dilutions of both bead and wash fractions were plated on SDCAA plates to quantify the selection yield. Two rounds of negative selection were performed with bare beads and beads coated with human polyclonal IgG, and yeast unbound to negative selection beads passed onto a round of positive selection with selection antibody-coated beads (PG9, PG16, PGT145 and 697-30D).

A final selection (resulting in generation 1.3) was performed using fluorescence activated cell sorting (FACS) with all steps performed in PBSF. The yeast library was stained with a combination of PG9 and 697-30D, each at a final concentration of 20 μg/mL, and a 1:200 dilution of chicken anti-c-myc (Gallus Immunotech) for detection of the C-terminal c-myc expression tag. After incubation with primary antibodies for 1 h at 4°C with rotation, yeast were washed three times and incubated with mouse anti-hu Fc-PE (Southern Biotech) and goat anti-chicken Alexa Fluor 647 (Life Technologies). Cells were washed twice, filtered through a 40 μm cell strainer (BD biosciences), and resuspended in 2 mL PBSF prior to sorting on an iCyt sy3200 system (Sony), selecting for antibody binding and expression tag signal.

### HEK surface display and flow cytometric analysis

Constructs evaluated in HEK cell membrane display were cloned into the pPPI4 vector (Sanders *et al.*, 2002) containing a C-terminal gpi anchor motif. Small-scale 2–5 mL HEK cell cultures were transfected with PEI as above. Cells were stained for flow cytometric analysis essentially as for yeast above; 1.5–2.5e5 cells were washed in PBSF three times prior to incubation in primary antibody at desired concentration and 1:200 mouse anti-HA (Biolegend) in 50 μL. Cells were washed three times, and stained with anti-hu Fd-FITC (ThermoFisher) at a 1:500 dilution and goat anti-mouse (H + L) Alexa Fluor 488 (Life Technologies); or with 1:1000 dilutions of anti-hu (H + L) Alexa Fluor 647 and anti-mouse (H + L) Alexa Fluor 488 (both Life Technologies); and a 1:40 dilution of calcein violet live-dead stain (ThermoFisher).

For analysis, cells were first gated on forward and side scatter, followed by gating on calcein violet for live cells. Cells expressing protein were gated using anti-HA antibody signal, and the resulting median fluorescence intensity was quantified as a measure of antibody binding.

## Results

### Recognition of a computationally designed and experimentally optimized single-chain V1V2 trimer by quaternary-preferring mAbs

With the goal of developing a V1V2-specific immunogen or probe with potential to elicit or detect quaternary-preferring bnAbs, the design objective for this study was to engineer a single-chain antigen with three copies of the V1V2 loops [sc-(V1V2)^3^] while maintaining native trimeric positions and conformation. To design the sc-(V1V2)^3^ such that the V1V2 loops would maintain their trimeric conformation and orientation, the full-length BG505 envelope trimer crystal structure ((Julien *et al.*, 2013a), PDB 4NCO) was used as a starting blueprint. Using a protein peeling algorithm (Gelly *et al.*, 2006; Gelly and de Brevern, 2011), the V1V2 loops were extracted as compact sub-domain protein units, resulting in V1V2 boundaries in concordance with other studies (HxB2 residues 126-196) (Kayman *et al.*, 1994; McLellan *et al.*, 2011). The resulting discontinuous V1V2 loops were re-ordered and re-wired for single-chain expression: new termini were selected based on minimization of residue-residue contacts, resulting in a circular permutation of the native V1V2 expression order and new termini in the hypervariable region of V2 to avoid disrupting secondary structure. Helical bridges appropriate for connecting the ends of the V1V2 loops to maintain their trimeric spacing were selected by the MASTER algorithm (Zhou and Grigoryan, 2015) from existing PDB structures (Fig. 1A).

Conformation of V1V2 epitopes in sc-(V1V2)^3^ expressed solubly in HEK293 cells was then evaluated by characterizing binding against a panel of human V1V2-specific mAbs recognizing distinct V1V2 conformations (Table I) using a customized bead-based immunoassay (Brown *et al.*, 2012). The sc-(V1V2)^3^ bound to CH58, a human mAb which recognizes a linear epitope and is known to bind V1V2 peptides, as was expected from V1V2 loop amino acid sequence (Liao *et al.*, 2013). More promisingly, the sc-(V1V2)^3^ construct also bound human mAbs 697-30D, which recognizes a discontinuous conformational epitope (Gorny *et al.*, 1994, 2012), and quaternary-preferring PG9 (McLellan *et al.*, 2011) (Fig. 1B, left), though binding to other V1V2 quaternary-specific mAbs (CH01, CH02 and PGT145) was not observed (not shown). As this assay lacks the resolution to determine individual binding contributions from each V1V2 loop, the sc-(V1V2)^3^ binding profile here is a composite measurement, and it is not known from this data whether the V1V2 loops in this design are antigenically equivalent. To place this composite sc-(V1V2)^3^ mAb binding profile in context with that for an existing V1V2 probe, a gp70 V1V2 AE.A244 probe—the V1V2 loops from clade AE fused to a portion of the murine leukemia virus gp70—which has previously been used to characterize V1V2-Ab responses (Kayman *et al.*, 1994; Haynes *et al.*, 2012), bound mAbs CH58 and 697-30D but not PG9. While a gp120 and a V1V2 tag from strain A244 have been shown to bind PG9 previously (Liao *et al.*, 2013), this gp70 V1V2 AE.A244 probe has also been shown to have much weaker binding to PG9 (Yates *et al.*, 2018), suggesting that the V1V2 loops do not fully retain their antigenicity in a gp70 scaffold context. Here, Ab binding to sc-(V1V2)^3^ and gp70 V1V2 AE.A244 cannot be directly compared, as construct and strain differences are likely to contribute to binding differences, yet the modest binding of PG9 to sc-(V1V2)^3^, which gp70 V1V2 AE.A244 lacks, suggests some propensity for proper quaternary V1V2 conformation in this construct which would be a requirement for an immunogen or probe to elicit or detect such Ab responses.

**Fig. 2:**
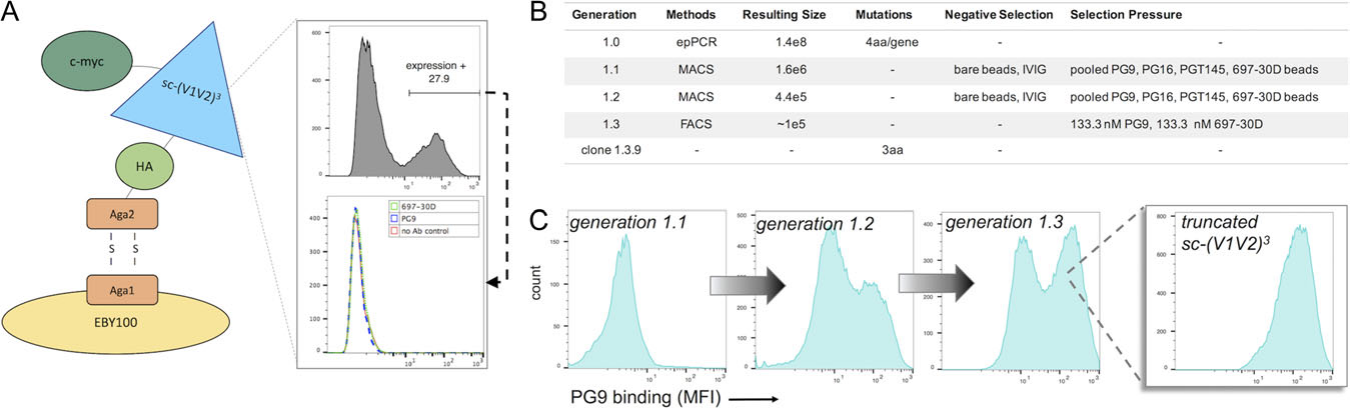
Yeast display and directed evolution of sc-(V1V2)^3^. (**A**) Schematic of yeast surface display (left) and initial expression of the wildtype sc-(V1V2)^3^. Yeast were gated for anti-c-myc signal (right, top), and this protein expressing population was assessed for antibody binding (right, bottom). (**B**) Summary of sc-(V1V2)^3^ library generation and selection strategy: one round of diversification via error-prone PCR was followed by two rounds of magnetic selection and one round of fluorescence activated cell sorting, enriching for clones with enhanced binding to selection antibodies. (**C**) Progression of PG9 binding over three library generations and characterization of a single over-represented clone from population 1.3, the truncated sc-(V1V2)^3^.

To explore further experimental optimization of epitope conformation, the sc-(V1V2)^3^ was then cloned into the yeast surface display system (Boder and Wittrup, 1997) as a C-terminal fusion to the yeast Aga2 mating protein, resulting in covalent linkage of the sc-(V1V2)^3^ to the yeast cell wall. The wildtype (WT) sc-(V1V2)^3^ showed expression in this system as measured by flow-cytometric detection of a C-terminal c-myc expression tag. However, lack of binding to 697-30D or PG9 indicated some loss of conformational fidelity in this expression system (Fig. 2A). Directed evolution of a yeast surface display library generated from one round of error-prone PCR via three rounds of magnetic- or fluorescence-activated cell sorting (Chao *et al.*, 2006; Ackerman *et al.*, 2009) using conformation-dependent and quaternary-preferring/specific mAbs as selection agents resulted in clones with enhanced binding to 697-30D and PG9 (Fig. 2B and C, Supplemental Fig. S1). PGT145 remained non-reactive despite being used as a selection reagent (not shown). Twelve clones from the selected population were sequenced, and of these, five clones with greatly enhanced binding to both 697-30D and PG9 were identical (full sequence in Supplemental Fig. S2). Curiously, this enriched clone, hereafter called ‘truncated sc-(V1V2)^3^’, was truncated after residue 88, which was presumed to have occurred during construction of the yeast library by homologous recombination due to the repetitive DNA sequences of the three V1V2 loop copies in the sc-(V1V2)^3^. With a truncation in the hypervariable region of V2-A (Fig. 1A, Supplemental Fig. S2), truncated sc-(V1V2)^3^ thus essentially consists of one complete V1V2 loop, comprised of the C-terminal end of one V1V2 loop (V2-Cb in Fig. 1) and the N-terminal end of the majority of a second V1V2 loop. The truncation in the isolated clone suggests that enhanced recognition by PG9 and 697-30D can be achieved in the context of a monomer. Nevertheless, the use of both computational protein design and directed evolution resulted in isolation of a truncated sc-(V1V2)^3^ variant with a phenotype of enhanced recognition by both quaternary-preferring and conformation-dependent mAbs suggesting desirable V1V2 loop conformational modifications.

### A K-M point mutation may stabilize V1V2 loop conformation

**Fig. 3:**
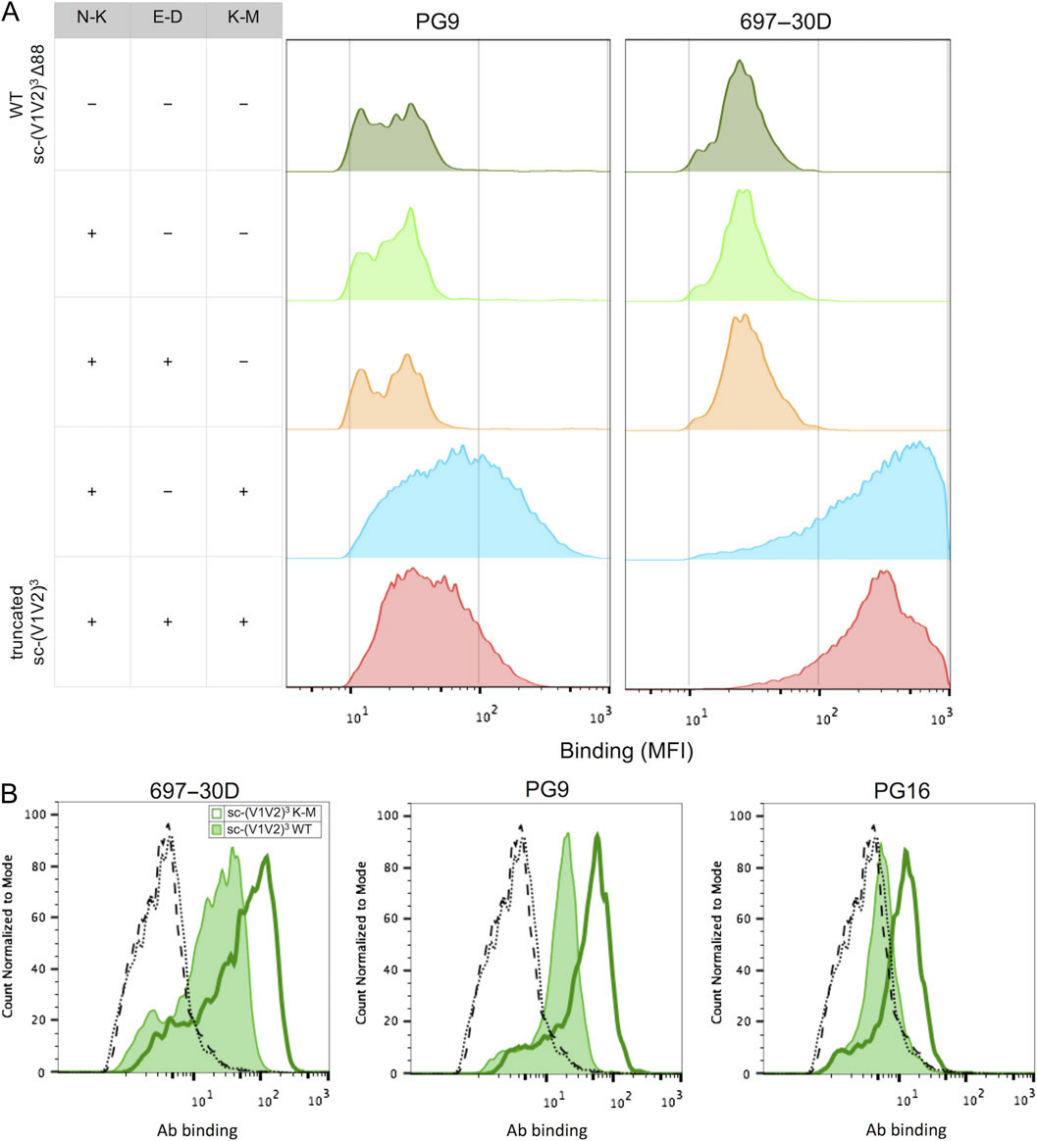
Identification of the K-M substitution is necessary and sufficient for enhanced Ab recognition in both yeast and HEK293 systems. (**A**) Histograms of median fluorescence intensities of expressing c-myc positive yeast for PG9 and 697-30D binding to a panel of yeast display truncated sc-(V1V2)^3^ revertants. (**B**) Histograms of 697-30D, PG9 and PG16 binding to WT (fill) and K-M (line) full-length sc-(V1V2)^3^ displayed on HEK cell membrane. Dashed and dotted lines represent no antibody controls for WT and K-M sc-(V1V2)^3^, respectively.

In comparison to the wildtype (WT) design, sequencing of the truncated sc-(V1V2)^3^ clone 1.3.9 revealed three amino acid mutations: N6K and K55M occurring in the V1V2 loop, and E31D in a helical bridge, hereafter referred to as N-K, K-M and E-D, respectively (Supplemental Fig. S2). To determine the phenotypic effect of each of these mutations, and whether this effect acts on the V1V2 loops, the helical bridge—i.e. stabilizing the truncated protein construct—or both, a panel of yeast-displayed truncated sc-(V1V2)^3^ revertants was generated. Staining with PG9 and 697-30D showed that the inclusion of the K-M substitution was necessary for enhanced binding to these mAbs (Fig. 3A). As expression host may be an important factor in eventual immunogen or probe production, especially with respect to relevant post-translational glycosylation, K-M was additionally introduced in the full-length sc-(V1V2)^3^ expressed as a gpi-anchored protein on the HEK cell membrane and resulted in enhanced binding to 697-30D, PG9 and PG16 (Fig. 3B). This result therefore demonstrated that the K-M mutation was sufficient to impart enhanced Ab recognition.

Promisingly, the K-M phenotype is observed in both yeast and mammalian expression platforms used here despite known differences in glycan processing: yeast proteins exhibit oligomannose glycoforms while mammalian proteins exhibit complex and hybrid glycoforms. Notably, immunogenicity of recombinant HIV envelope proteins may depend on variable glycosylation with respect to quantity and quality (Go *et al.*, 2008), and Abs can also have different glycan preferences. Consistent with these differences in glycosylation between expression hosts, introduction of K-M in HEK293 displayed sc-(V1V2)^3^ also enhanced PG16 binding. This result, which was not observed in the yeast expression system (data not shown), may reflect PG16's requirement for a sialylated hybrid moiety at N173 (Pancera *et al.*, 2013).

**Fig. 4:**
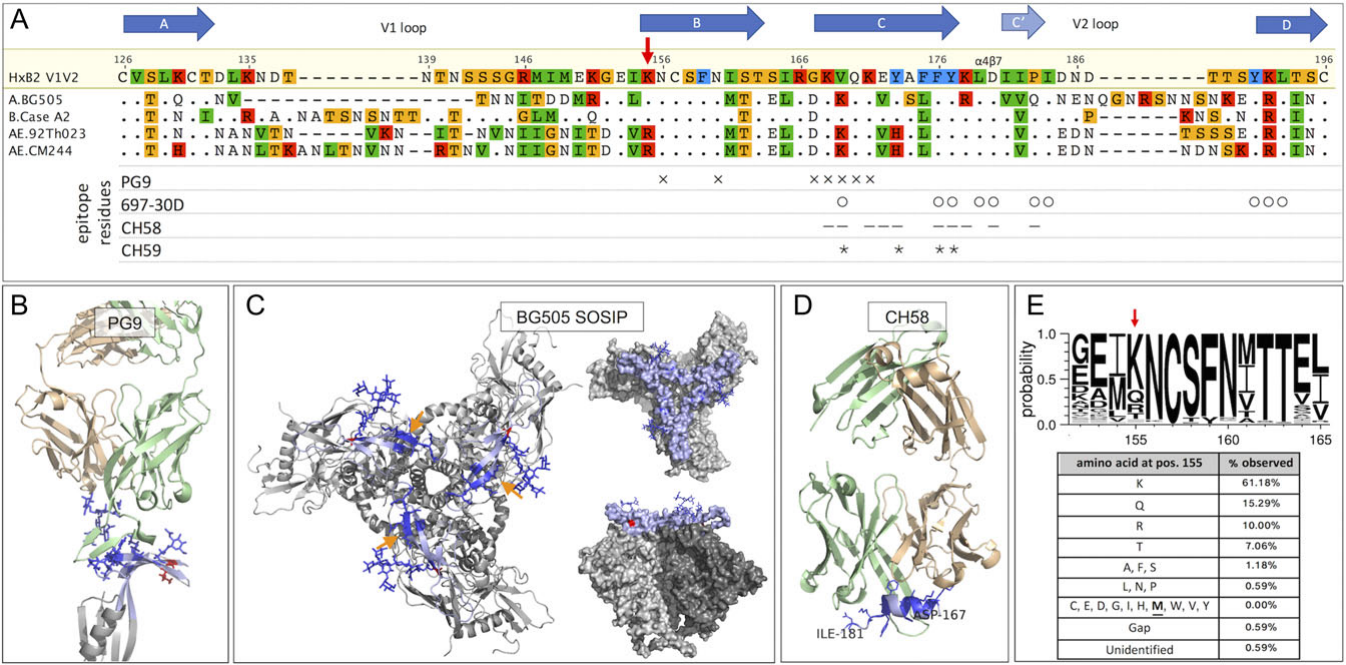
The K-M substitution in the context of HIV-1 V1V2 sequence and structure. **(A**) Sequence alignment of V1V2 from four strains to HxB2. Secondary structure elements (McLellan, *et al.*, 2011) are depicted with arrows above. Known epitope residues defined by mutagenesis or crystallography (697-30D; PG9, CH58, CH59) are denoted with symbols below. (**B**) PG9 Fab complexed with scaffolded V1V2 (McLellan, *et al.*, 2011). V1V2 loops are shown in light blue; PG9 contact residues and glycans are shown in dark blue. Lysine at the K-M location (K155) is highlighted in red, with side-chain pointing away from PG9 Fab. (**C**) PG9 contact residues (dark blue) and K155 (red) in the context of the full-length BG505 SOSIP trimer in cartoon (left) and space-fill (right-top: top-view; right-bottom: side-view) representations (Julien *et al.*, 2013a). (**D**) CH58 in complex with its cognate peptide in a coil-helix conformation (Liao *et al.*, 2013). Coil-helix peptide region in CH58-bound form corresponds to the C-strand in the full trimer structure (arrows in B). (**E**) Sequence logogram with position 155 marked by arrow above and tabulation of amino acid usage in the V1V2 loops.

Placing the K-M mutation in the context of known V1V2 sequences and available crystal structures suggests a phenotype that may affect the overall conformation of the V1V2 loops. The K-M substitution identified in the truncated sc-(V1V2)^3^ clone corresponds to position 155 in the HxB2 numbering scheme (K155 in clades A and B; R155 in clade AE), and is located in a well-conserved region at the N-terminus of the β-strand of the Greek-key motif assumed by the V1V2 loops in complex with PG9 (McLellan *et al.*, 2011) (Fig. 4A). Though K-M is located near key PG9 contact residues, structural details suggest that the phenotypic effect of K-M may have an overall conformational effect rather than a PG9-specific effect: in the context of the full-length envelope trimer, K-M occurs on the underside of the V1V2 loops directed away from the N156/N160 glycans required for PG9 binding (Fig. 4B), and its side-chain is inaccessible from the trimer crown at which PG9 is directed as demonstrated in a surface rendering (Fig. 4C, insets) (Julien *et al.*, 2013b). To complement this picture, mAb 697-30D is known to require contact residues distinct from those used by PG9 as determined by alanine scanning (Fig. 4A) (Gorny *et al.*, 2012), and thus, the effect of K-M extends to enhanced 697-30D binding despite distinct epitope residues. Additionally, it has been suggested that 697-30D requires glycan-mediated stabilization of the V1V2 loops (Gorny *et al.*, 1994), and N/Q substitutions in V1V2 constructs reduce but do not abrogate 697-30D binding (Liao *et al.*, 2013). Accordingly, as measured in a customized bead-based immunoassay, binding was reduced for a deglycosylated gp70 V1V2 BG505 WT construct, while deglycosylation of the corresponding K-M variant did not reduce 697-30D binding suggesting glycan-independent stabilization by the K-M substitution (Supplemental Fig. S3). In contrast to PG9 and 697-30D, mAbs CH58 and CH59 are known to bind a V1V2 peptide (residues 167–181) which assumes helix-coil secondary structures when bound to these mAbs (Fig. 4D) (Liao *et al.*, 2013). Interestingly, this peptide assumes a β-strand in the PG9-bound and trimeric V1V2 structures (orange arrows in Fig. 4C), and thus, the V1V2 conformation recognized by PG9/697-30D may be mutually exclusive with CH58/CH59 binding. Furthermore, that a methionine is not frequently observed at position 155 in known HIV-1 sequences (Fig. 4E) suggests that this mutation may also have a functional effect on viral fitness beyond an antigenic effect. Therefore, the position of K-M relative to known epitope contacts; the phenotypic effects on both trimer-preferring PG9 and conformation-dependent 697-30D binding, and perhaps on linear epitope-specific CH58/CH59 recognition; and sequence information indicating a possible effect on viral fitness, thus suggest that K-M may result in a V1V2 conformational modification such as a stabilization of the β-strand involved in the super-secondary structure found in the full prefusion envelope trimer and the PG9 epitope.

PG9 recognition is highly dependent on the orientation and conformation of the V1V2 loops, which may be impacted by molecular context. In monomeric gp120, the V1V2 loops may freely pivot with respect to the remainder of the molecule, and it is possible that formation of the full trimer makes a key contribution to constraining and optimally orienting the V1V2 loops for PG9 recognition (Walker *et al.*, 2009; McLellan *et al.*, 2011). Thus, the K-M substitution may constrain the V1V2 loops in a way that mimics their β-stranded trimeric conformation in the prefusion trimer. Indeed, when K-M was introduced in a HEK293 membrane-displayed BG505 SOSIP gp140 with a native flexible linker, which trimerizes in this context (Grundner *et al.*, 2002; Sharma *et al.*, 2015), a less dramatic increase in PG9 and PG16 binding was observed, perhaps indicating that K-M is unnecessary for V1V2 loop conformational fidelity in the context of an already stabilized trimer construct (Supplemental Fig. S4).

### K-M confers an antigenic phenotype extending to multiple HIV-1 strains

**Fig. 5:**
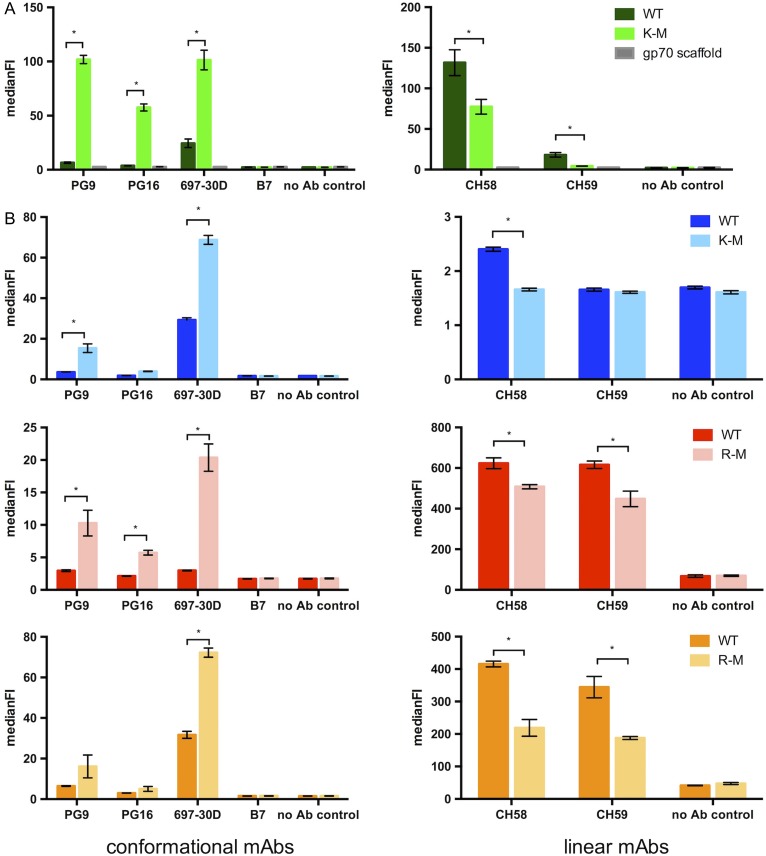
Altered mAb binding to monomeric gp70 V1V2 K-M across HIV-1 strains. (**A**) Comparison of binding of trimer-preferring/conformation-dependent (left) and peptide-binding (right) mAbs to HEK cell membrane-displayed gp70 V1V2 A.BG505 WT or K-M as measured by flow cytometry. (**B**) Comparison of binding of trimer-specific/conformation-dependent (left) and peptide-binding mAbs (right) to HEK cell surface-displayed gp70 V1V2 WT and K/R-M variants in three strains. Significance was calculated in Prism GraphPad, using multiple *t* tests with the Holm–Sidak method, alpha = 5%. Error bars represent standard deviation of triplicate measurements.

To confirm that the K-M phenotype was localized to the V1V2 loops as opposed to an effect specific to the truncated or full-length sc-(V1V2)^3^ design, the V1V2 loops from the reference strain BG505 were cloned as a C-terminal fusion to a monomeric gp70 scaffold (gp70 V1V2 BG505) and expressed on the HEK cell membrane as above. Similarly to the sc-(V1V2)^3^ K-M, gp70 V1V2 BG505 K-M showed increased binding to mAbs PG9, PG16 and 697-30D (Fig. 5A) suggesting again that the K-M phenotypic effect is not specific to sc-(V1V2)^3^, but instead may act generally in the context of a monomeric V1V2 loop. Notably, this result also demonstrates that, while the quaternary-preferring nature of PG9 and PG16 may result from quaternary contacts with V1V2 glycans on neighboring protomers in the envelope trimer, (Julien *et al.*, 2013b), the enhanced binding to the K-M variant is observable even in this monomeric V1V2 context. Given previous studies suggesting that the V1V2 loops may have conformational differences in monomeric gp120 as compared to β-strands found in the envelope trimer (Liao *et al.*, 2013), the result here further suggests a β-strand stabilizing effect of this substitution.

Considering the K-M phenotype beyond quaternary-preferring and conformation-dependent mAb binding, we tested the hypothesis suggested by the CH58:V1V2 and CH59:V1V2 peptide crystal structures (Liao *et al.*, 2013) that, with enhanced PG9 and 697-30D binding, K-M may promote a conformation incompatible with CH58 and CH59 binding. Indeed, for the gp70 V1V2 BG505 monomer, CH58 and CH59 showed decreased binding to the K-M mutant as compared to WT (Fig. 5A, right), even though the selection strategy employed in directed evolution of the sc-(V1V2)^3^ (Fig. 2B) did not include negative selection against recognition by these linear epitope mAbs.

The effect of K-M on multiple classes of mAbs and its position in a region of conserved secondary structure (Fig. 4A), further suggested a conformational phenotype that may extend beyond strain BG505. In addition to the gp70 V1V2 BG505, we expressed gp70 V1V2 from strains B.Case_A2 (clade B), AE.92Th023 and AE.CM244 (both clade AE) with and without the K-M mutation using mammalian cell membrane display. Binding differences between WT and K-M gp70 V1V2 for each of these strains largely recapitulated those observed for gp70 V1V2 BG505, with overall enhanced binding to PG9, PG16 and 697-30D and reduced binding to CH58 and CH59 for the K-M mutant constructs (Fig. 5B). Notably, as CH58 and CH59 were isolated from an RV144 vaccinee receiving the AIDSVAX B-E boost containing gp120 from clade AE (Rerks-Ngarm *et al.*, 2009; Liao *et al.*, 2013), differences in binding to gp70 V1V2 AE.92Th023, AE.CM244 WT vs. R-M mutants were only resolved when stained at a lower mAb concentration (5.55 nM for gp70 V1V2 from clades AE vs 66.7 nM for others; medianFIs are not comparable those for B.CaseA2, left, as a different anti-human secondary reagent was used). That the phenotypic effect of this K-M mutation is transferrable across HIV strains again points to a conformational modification in structurally conserved elements of the V1V2 loops, and may explain the enhanced binding by a bnAb like PG9, which gains its breadth via recognition of conserved super-secondary structure elements and contacts to the V1V2 peptide backbone (McLellan *et al.*, 2011).

### V1V2 K-M variants have potential utility to dissect conformational V1V2 humoral responses

**Fig. 6:**
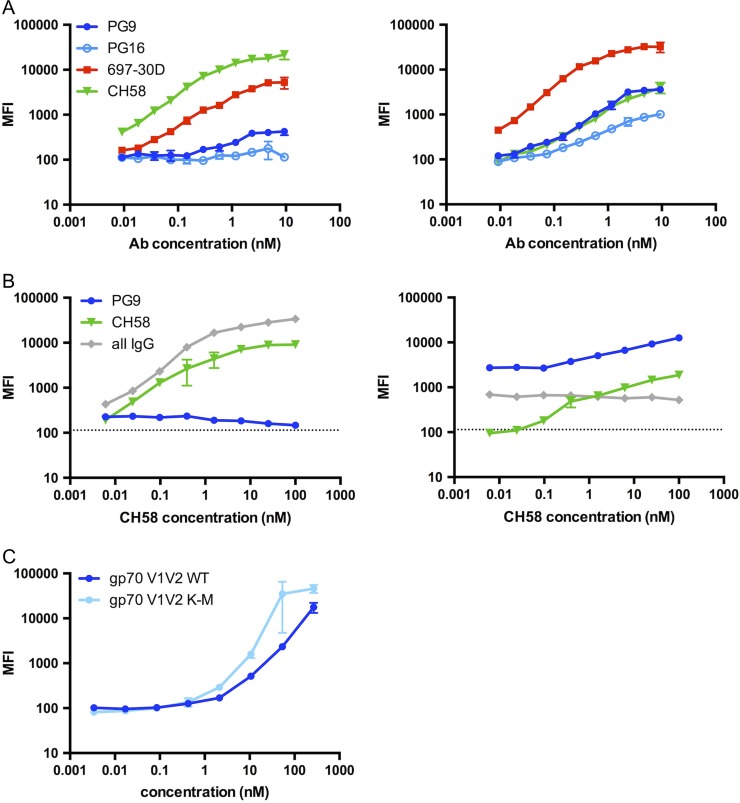
Soluble gp70 V1V2 K-M variants as potential epitope-specific probes. (**A**) Binding profiles of soluble gp70 V1V2 WT and gp70 V1V2 K-M to peptide-binding (CH58), conformation-dependent (697-30D) and quaternary-preferring mAbs (PG9 and PG16). B7 is included as an anti-Dengue negative control. (**B**) Reactivity of gp70 V1V2 WT (Left) and gp70 V1V2 K-M (Right) to CH58, PG9 and total IgG in a mAb mixture: titration of CH58 over constant PG9 concentration (16.7 nM). Dashed lines correspond to background signal for each detection reagent, α-human kappa, α-human lambda and α-human IgG for PG9, CH58 and all IgG, respectively. (**C**) Binding profiles of gp70 V1V2 constructs to HIVIG (pooled HIV Ig). Error bars represent standard deviation of triplicate measurements.

Given the value of a more nuanced understanding of V1V2 Ab recognition with respect to epitope conformation, we explored the utility of a K-M variant to discern mAb epitope conformation specificity. Because the gp70 V1V2 WT probes show little observable binding to quaternary-preferring bnAb PG9 (Fig. 5), PG9-like responses may fail to be reliably detected given currently available V1V2-based probes. Therefore, the enhanced reactivity of conformational mAbs to gp70 V1V2 K-M variants may make such variants more suitable probes for examining humoral responses raised against these conformational epitopes. Both gp70 V1V2 BG505 WT and K-M were secreted from mammalian cells and their potential to discern conformational preferences of Ab binding was compared by measuring relative quaternary-preferring/conformational/linear mAb binding. Each antigen exhibited different binding profiles to CH58, 697-30D, PG9 and PG16: WT gp70 V1V2 showed strongest binding to CH58, intermediate binding to 697-30D, and minimal binding to PG9 and PG16 (Fig. 6A, left); the K-M variant showed an altered antigenicity profile, with strongest binding to 697-30D, modest binding to PG9 and PG16, and markedly decreased binding to CH58 relative to the WT gp70 V1V2 (Fig. 6A, right). The distinct antigenicity profiles thus suggest that gp70 V1V2 K-M would be more specific than WT for V1V2-targeting conformational antibodies, as it displays the decreased cross-reactivity to CH58.

Beyond recognition by mAbs, a useful probe must have the ability detect different conformational Ab responses within a polyclonal mixture in subject sera. To determine whether the above probes would detect a rare PG9-like trimer-preferring response within a simultaneously present linear CH58-like response, the individual contributions of PG9 and CH58 binding were measured in a mixture of the two mAbs, keeping PG9 constant at a 16.7 nM concentration over a CH58 titration curve. The gp70 V1V2 WT showed minimal binding to PG9, and a dose-dependent response to CH58, which was likely the main contributor to total IgG binding (Fig. 6B, left). For the gp70 V1V2 K-M probe, binding to PG9 was constant given its constant concentration, binding to CH58 was decreased relative to the WT probe, and total IgG binding to the K-M variant represented additive contributions from CH58 and PG9 (Fig. 6B, right). Thus, the gp70 V1V2 K-M probe is sensitive and specific for conformational Ab responses, where the WT probe is not.

As further confirmation of the utility of K-M variants to dissect humoral responses to V1V2 conformational epitopes, reactivity to pooled immunoglobulin from asymptomatic HIV-infected individuals (HIVIG, NIHARP Cat #3957) was measured with this panel of probes (Fig. 6C). Mutant gp70 V1V2 K-M showed higher reactivity than WT, indicating that it is perhaps sensitive to a different set of Abs within this immunoglobulin mixture. The qualitative differences between the Abs reactive to gp70 V1V2 K-M vs. WT remain to be examined, and could be accomplished with antigen-specific Ab purification using these probes as bait.

## Discussion

In this study, we demonstrate the use of computational structure-based protein design, and directed evolution towards development of an HIV-1 V1V2 antigen that faithfully presents conformational epitopes recognized by bnAbs. The resulting HIV-1 V1V2 K-M variant isolated and described here is recognized by trimer-preferring and conformational mAbs PG9, PG16 and 697-30D, suggesting structural recapitulation of the β-stranded V1V2 conformation present in the prefusion envelope trimer. Application of the K-M substitution in V1V2 immunogens or probes may enable elicitation or dissection of conformation-dependent humoral responses directed at the V1V2 loops in settings of vaccination and infection.

This work demonstrates the utility of yeast surface display and directed evolution as a strategy to stabilize epitopes that may otherwise lose their conformation in epitope-focusing or specific constructs. While a limitation of yeast expression of mammalian proteins is their differential glycosylation, yeasts’ restriction to oligomannose glycoforms may not have been a disadvantage in this study as PG9, a mAb used for selection, displays a dependency on a mannose moiety at N160 in the V1V2 loops (McLellan *et al.*, 2011). More generally, however, glycosylation profiles may dramatically affect some Ab binding (e.g. in this study, PG16, which requires a sialylated hybrid glycan at N156 (Pancera *et al.*, 2013), bound mammalian-expressed K-M variants, but not those expressed in yeast), and as such, the glycosylation profile of the expression system used, either during directed evolution or subsequent protein production, should be considered and manipulated where necessary. Regardless, the mAbs used here (PG9 and 697-30D) tolerate the glycosylation differences between yeast and the HEK293 platforms used, and the relative phenotypic differences between WT and K-M constructs are consistent across expression platforms, suggesting a glycan-independent effect.

We have shown that a K-M substitution may have an effect on V1V2 conformation, and it may be important in the case of HIV-1 V1V2 immunogen design to achieve proper V1V2 loop conformation in a reductionist, epitope-focused probe or immunogen. We have also demonstrated the potential utility of K-M variants in characterizing sera with finer resolution towards conformational specificity of humoral responses. Current commonly used V1V2-specific probes such as the gp70 V1V2 B.Case A2 used in characterizing RV144 trial participant sera (Haynes *et al.*, 2012) may more readily detect linear Ab responses, and as such would give poor readouts for conformational humoral responses. That the WT and K-M variant in gp70 V1V2 constructs show different binding profiles to distinct classes of mAbs and to HIVIG suggests that these probe variants may react with distinct sub-populations of V1V2-specific Abs, which could be useful in identifying sera in which conformational V1V2 responses are dominant or selecting patient/vaccinee samples from which conformational mAbs are more likely to be isolated. Furthermore, the K-M substitution and the single-chain design approach initially used for the sc-(V1V2)^3^ could aid design of other next-generation antigen probes and immunogens such as BG505 SOSIP.664 gp140 (Sok *et al.*, 2014) and trimeric or multimeric scaffolded V1V2s (Gorman *et al.*, 2016; He *et al.*, 2016; Jiang *et al.*, 2016). These constructs have been designed with the goal of isolating, characterizing, or eliciting quaternary-specific V1V2 bnAbs, but a truly trimeric epitope-focused antigen has yet to be engineered. While BG505 SOSIP.664 gp140 recapitulates quaternary epitopes, as a probe it lacks epitope specificity. Additionally, though three scaffolded copies of the V1V2 loops were present in the constructs by Gorman *et al.*, these failed to mimic the oligomeric configuration present in the prefusion trimer. Adding to this arsenal of design approaches, K-M could stabilize the V1V2 loops as a single point mutation without needing to screen for scaffolds to stabilize the V1V2 β-stranded secondary structure (McLellan *et al.*, 2011; Jiang *et al.*, 2016). Thus, this work may contribute to further development of antigens for use as probes characterizing sera or baits for B cell isolation or as immunogens. Furthermore, though the sc-(V1V2)^3^ was not shown here to bind quaternary-specific mAbs like PGT145, further directed evolution of this single-chain design could be pursued to accomplish better quaternary epitope mimicry.

Finally, we sought to push the boundaries of minimal antigen/immunogen engineering. Though the initial goal to mimic quaternary conformation with the sc-(V1V2)^3^ was not met, identification of the K-M substitution may represent a modification resulting in preserved antigenicity of the V1V2 loops that could be incorporated in other antigen designs. Others have developed small V1V2 constructs, such as the V1V2-tags containing little more than a leader sequence and purification tags (Liao *et al.*, 2013), and gD V1V2 which consists of the V1V2 loops scaffolded on the first 27 residues of the Herpes Simplex Virus gD protein (Morales *et al.*, 2016). The K-M substitution may improve the V1V2 antigenicity profile in the contexts of constructs like these as well. Therefore, successful design of such minimal antigens for next-generation immunogens will not only have the benefit of excluding extraneous protein scaffolds, but they may also afford unprecedented conformational specificity of epitope presentation, which may be critical for metastable viral fusion glycoproteins for which only a subset of conformations are relevant to Ab recognition and neutralization.

## Supplementary Material

Supplementary Data
