# Supplementary material for: Towards conformational fidelity of a quaternary HIV-1 epitope: computational design and directed evolution of a minimal V1V2 antigen
Source: Protein Eng Des Sel. 2018 Jun 12;31(4):121–33. doi: 10.1093/protein/gzy010 (PMC6030936; doi:10.1093/protein/gzy010)
Supplement: Supplementary Data [file gzy010jilpedssupplementalr2.pdf]

Supplementary Information

Towards conformational fidelity of a quaternary HIV-1 epitope: computational design and directed evolution of a minimal V1V2 antigen

Jennifer I. Lai[1], Deeptak Verma[2], Chris Bailey-Kellogg[2], Margaret E. Ackerman[1,3,*]

**Supplemental Figures**	
Supplemental Figure 1	FACS plots for sorting of sc-$(V1V2)^3$ yeast surface display library generation 1.2 against PG9 and 697-30D
Supplemental Figure 2	Wildtype sc-$(V1V2)^3$ and truncated sc-$(V1V2)^3$ sequence alignment
Supplemental Figure 3	Deglycosylation of gp70 V1V2 does not result in loss of phenotype
Supplemental Figure 4	K-M substitution in gp160 A.BG505 SOSIP in HEK cell membrane display

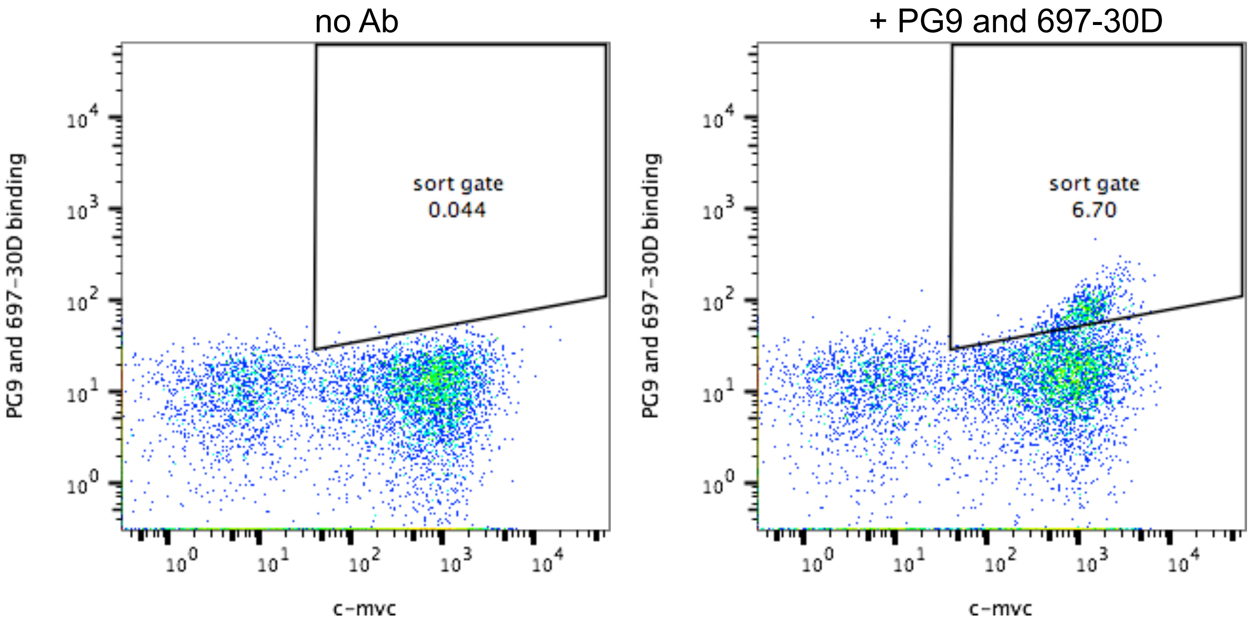

Supplemental Figure 1.

FACS plots for sorting of sc-(V1V2)^3 yeast surface display library generation 1.2 against PG9 and 697-30D. Sort gate was drawn based on a no primary Ab control. Cells collected from the sort gate were plated, and twelve clones were sequenced.

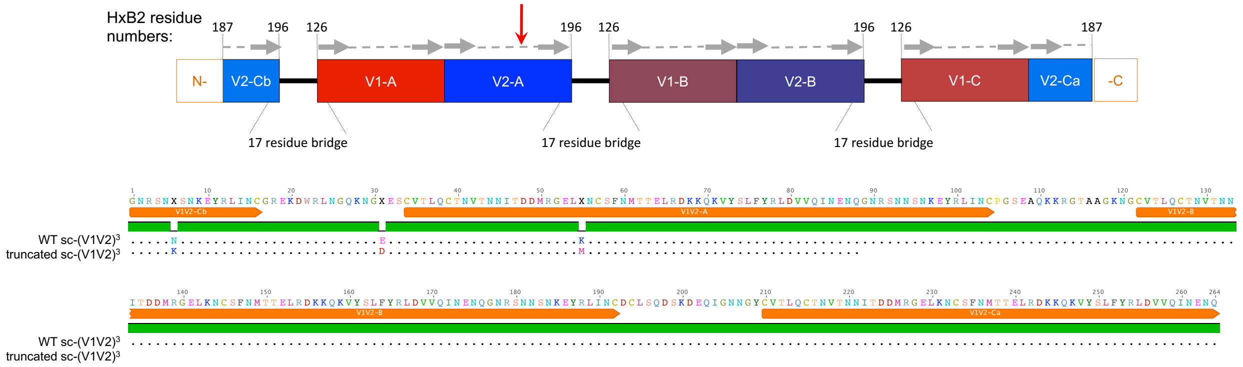

Supplemental Figure 2.

Wildtype sc-(V1V2)^3 and truncated sc-(V1V2)^3 sequence alignment. (Top) Sequence schematic of sc-(V1V2)^3 as in Figure 1, with the location of the residue 88 truncation depicted with a red arrow. (Bottom) Sequence alignment of sc-(V1V2)^3 and the isolated truncated sc-(V1V2)^3. V1V2 loops are annotated in orange (V1V2-A, V1V2-B, V1V2-Ca/V1V2-Cb) as in the schematic above.

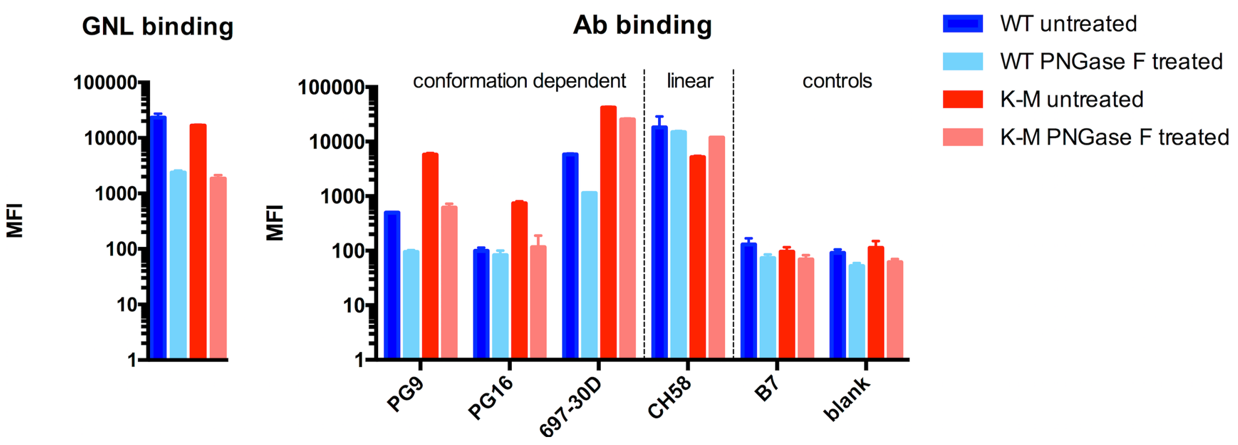

Supplemental Figure 3.

Deglycosylation of gp70 V1V2 does not result in loss of K-M phenotype. Multiplex assay beads conjugated with gp70 V1V2 A.BG505 WT and gp70 V1V2 A.BG505 K-M were treated with PNGase F. Deglycosylation was measured by binding to biotinylated Galanthus Nivalis Lectin (left). Binding was measured to PG9 and PG16 (glycan-dependent) as well as to 697-30D, CH58, and anti-Dengue B7. Error bars represent standard deviation of triplicate measurements.

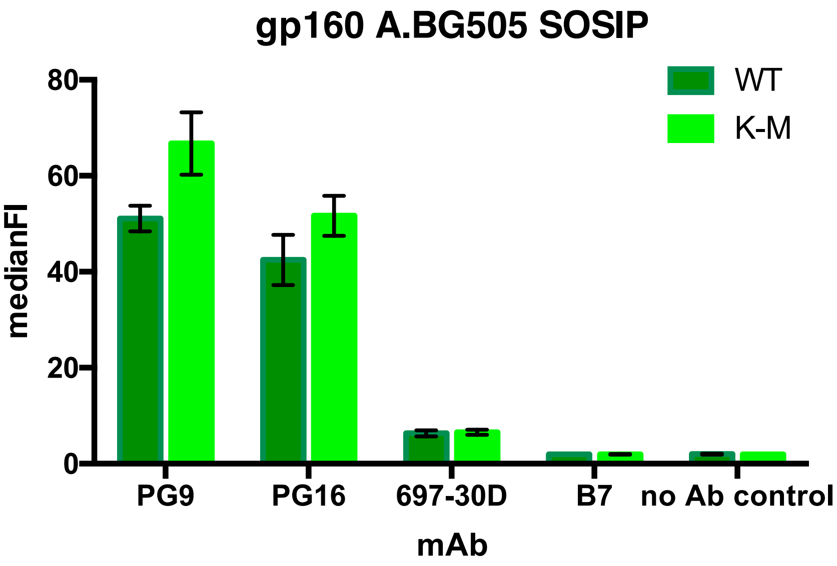

Supplemental Figure 4.

K-M substitution in gp160 A.BG505 SOSIP in HEK cell membrane display. Error bars represent standard deviation of triplicate measurements.
